# Actinomycosis in a gray four-eyed opossum (*Philander opossum)* caused by a novel species of *Schaalia*

**DOI:** 10.1186/s12917-021-02937-3

**Published:** 2021-07-13

**Authors:** Stefanie Knoepfler, Alexandria Schauer, Andreas Thomann, Simon Feyer, Peggy Rüegg-van den Broek, Olivier Jean Glardon, Sonja Kittl

**Affiliations:** 1 Institute of Veterinary Bacteriology, Vetsuisse Faculty, University of Bern, 122 Laenggassstrasse, CH-3012 Bern, Switzerland; 2 Institute of Animal Pathology, Vetsuisse Faculty, University of Bern, 122 Laenggassstrasse, CH-3012 Bern, Switzerland; 3 Papiliorama Foundation, Moosmatte 1, CH-3210 Kerzers, Switzerland

**Keywords:** Gray four-eyed opossum (*Philander opossum)*, lumpy jaw disease, *Schaalia*, actinomycosis

## Abstract

**Background:**

Infective lesions of the jaws and adjacent tissues (lumpy jaw disease, LJD) have been recognized as one major cause of death of captive macropods. *Fusobacterium necrophorum* and *Actinomyces* species serve as the main source of LJD in kangaroos and wallabies. Currently, little is reported about LJD or similar diseases in opossums.

**Case presentation:**

Here we report a case of actinomycosis resembling the entity lumpy jaw disease in a gray four-eyed opossum, caused by a novel species of *Schaalia*. A 2.8 year old male *Philander opossum* was presented with unilateral swelling of the right mandible. After an initial treatment with marbofloxacin, the opossum was found dead the following day and the carcass was submitted for necropsy. Postmortem examination revealed severe mandibular skin and underlying soft tissue infection with subsequent septicemia as the cause of death. Histological examination demonstrated Splendore-Hoeppli phenomenon, typically seen in classical cases of actinomycosis. Bacteriology of liver and mandibular mass yielded a previously undescribed species of *Schaalia*, whose 16 S rRNA gene sequence was 97.0 % identical to *Schaalia canis*. Whole genome sequencing of the opossum isolate and calculation of average nucleotide identity confirmed a novel species of *Schaalia*, for which no whole genome sequence is yet available.

**Conclusions:**

The herewith reported *Schaalia* infection in the gray four-eyed opossum resembling classical actinomycosis gives a novel insight into new exotic animal bacterial diseases. *Schaalia* species may belong to the normal oral microbiome, as in macropods, and may serve as a contributor to opportunistic infections. Due to the lack of current literature, more insights and improved knowledge about *Schaalia* spp. and their pathogenicity will be useful to choose appropriate therapy regimens and improve the treatment success rate and outcome in exotic and endangered species.

## Background

The family of the *Actinomycetaceae* is comprised by a heterologous group of anaerobic or facultative anaerobic, asporogenous, non-acid-fast, filamentous or diphtheroid, rod-shaped organisms with a high DNA G + C content [1]. Among the most relevant genera in veterinary medicine are *Actinomyces, Schaalia, Actinobaculum* and *Trueperella.*[2].

The genus *Actinomyces* is one of the largest genera within the *Actinomycetaceae* [3].

*Actinomyces* species are ubiquitous, and can be found in soil and in the microbiota of animals and humans. While certain species are commensal in the skin flora and on mucus membranes, others are major pathogens, leading to intrinsic infections in humans and animals. Severe diseases in veterinary medicine are mostly associated with *A. bovis, A. viscosus, A. hordeovulneris* and *A. hyovaginalis*, whereas *A. israelii* is considered the primary pathogen affecting humans [2]. The causative *Actinomyces* species typically gain access to deeper tissue through trauma, surgical procedures or foreign bodies, which compromise the mucosal barrier. Within different tissues, the bacteria form masses consisting of aggregates of branching, filamentous bacilli and lead to chronic, pyogranulomatous reactions [4].

Other than fimbriae, peptidoglycan and biofilm production, as described in human cases, relatively little is known about virulence traits in the genus *Actinomyces* [5]. Specific virulence factors of *Actinomyces* species of veterinary importance have not yet been identified [2].

*Actinomyces* species are fastidious and thus not easy to culture and isolate. They grow slowly on sheep blood agar at 37 °C under capnophilic and/or anaerobic conditions. Colonies are grossly visible in approximately two days and appear mostly very small, with a maximum diameter of one millimeter. Furthermore, they are generally non-hemolytic, white, rough or smooth and can adhere tenaciously to solid medium. Within Gram-stained smears, bacteria are Gram-positive and appear as slightly branched filaments or short forms. On subculture, the bacteria may become diphtheroidal or coccobacillary [2].

Demonstration of Gram-positive-staining, filamentous organisms and sulfur granules on histological examination is strongly suggestive of a diagnosis of actinomycosis. However, isolation and identification of *Actinomyces* by conventional methods is often difficult and time consuming. The sequence analysis of the 16S rRNA gene and, in recent years, matrix-assisted laser desorption/ionization time-of-flight mass spectrometry (MALDI-TOF-MS) has become a rapid and simple method to identify *Actinomyces* species [6, 7].

16S rRNA gene sequencing has shown the genus *Actinomyces* to be very heterogeneous and phylogenetically intermixed with other genera such as *Arcanobacterium* and *Mobiluncus* [8, 9]. In 2018, some of the species were, according to the phylogenomic data, emended to the genera of *Bowdenia, Boudabousia, Buchananella, Gleimia, Pauljensenia, Schaalia* and *Winkia* [10]. This also applies to *Actinomyces canis*, first isolated from skin and mucous membranes of three dogs, which has been reclassified to the new genus *Schaalia* [10, 11]. Currently, the genus *Schaalia* is comprised of 12 published species (www.lpsn.dsmz.de, accessed 08.07.2020).

Here we report a case of actinomycosis caused by a novel species of the genus *Schaalia.* The species was isolated from a subcutaneous mandibular mass, liver and lung of a gray four-eyed opossum (*Philander opossum*). The gray four-eyed opossum is one of many opossum species in the order *Didelphimorphia* and the family *Didelphidae*. *P. opossum* is a neotropical marsupial, ranging in distribution from northeastern Mexico to southeastern Brazil. The habitat includes mainly tropical forest areas, such as tropical evergreen, secondary growth and gallery forests. Gray four-eyed opossums are primarily nocturnal, omnivorous and have a life span of up to 2.5 years and 3.5 years in the wild and in captivity, respectively [12, 13].

Not only is information regarding disease processes in the gray four-eyed opossum scarce, insights regarding *Schaalia* spp. infections and their pathogenicity is, to our knowledge, nonexistent. In other marsupial families (including kangaroos and wallabies), progressive pyogranulomatous osteomyelitis involving the mandible or maxilla (lumpy jaw disease, LJD) is a common bacterial disorder and is one of the most significant causes of illness and death in captive macropods [14]. The jaw infection is characterized by swelling around the face and jaw, sometimes involving mandibular lymph nodes and salivary glands. *Fusobacterium necrophorum* serves as the main source of LJD, whereas *Actinomyces* spp. and *Corynebacterium* spp. are less commonly isolated bacterial agents [15]. A bacteremia may result in systemic disease, disseminating organisms to other organs, including liver and lungs, as seen in the presently described case [15]. We report the isolation of a new species of *Schaalia* associated with mandibular skin and soft tissue infection, resulting in septicemia and death in a gray four-eyed opossum.

## Case presentation

**Fig. 1:**
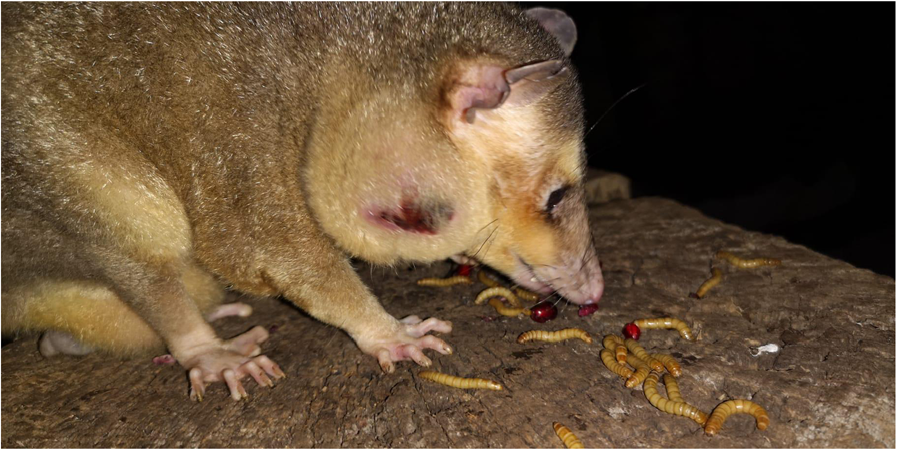
Male *Philander opossum* with unilateral ulcerated swelling of the right mandible

The 2.8 year old male *Philander opossum* was housed in a group of five members, including two parents and three offspring in an indoor enclosure in a zoo near Bern, Switzerland, and served as the sire of the group. The group had been gifted to the zoo two years before and had been living there ever since. The animal was presented to the zoo veterinarian for evaluation of a unilateral swelling in the area of the right mandible (Fig. 1), which eventually developed ulceration. The opossum was otherwise clinically sound. After ulceration occurred, initial treatments were performed that consisted of rinsing and local disinfection, as well as administration of an oral anti-inflammatory agent (meloxicam, 0.02 mg/kg, started several days later). Although the swelling appeared to ameliorate, the wound never fully closed, three weeks after initial treatments, oral antibiotic therapy consisting of marbofloxacin at a dose of 2.6 mg/kg was initiated. The next day, the opossum was found dead, despite having shown no additional premonitory clinical signs.

**Fig. 2:**
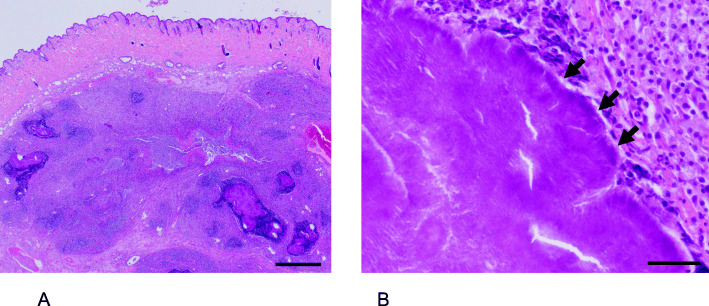
Histopathological examination of the subcutaneous mass, stained with hematoxylin and eosin. **A** Low magnification demonstrating characteristic pyogranulomatous inflammation. Scale bar = 1 mm. **B** Higher magnification, demonstrating dense mats of filamentous bacterial colonies (arrows) embedded in radiating, brightly eosinophilic material (Splendore-Hoeppli phenomenon). Scale bar = 50 μm

The carcass was submitted to the Institute of Animal Pathology at the University of Bern for postmortem examination. On gross examination, a focally extensive swelling of 3 × 2.5 × 1.5 cm was present on the caudolateral aspect of the mandible with a superficial, focal ulceration of 1.3 × 1.0 cm. On cut section, the swelling was located subcutaneously and was comprised of a well-demarcated, encapsulated, non-movable mass with an inhomogeneous, firm to friable, white to yellow cut surface. Histopathological examination demonstrated central areas of necrosis in addition to large, multifocal accumulations of intensely eosinophilic material arranged in radiating, club-like structures (Splendore-Hoeppli phenomenon), admixed with abundant filamentous bacterial rods (Fig. 2). This material was surrounded by high numbers of neutrophils, surrounded by macrophages, few multinucleated giant cells, and a thick, fibrous connective tissue capsule, characteristic of a pyogranuloma. Samples of other visceral organs, including lung, spleen, liver, and kidney revealed acute, neutrophilic inflammation, consistent with septicemia. Furthermore, there was abundant perivascular deposition of amorphous, extracellular, acellular, pale eosinophilic material, which displays green birefringence following polarisation in Congo-red staining (compatible with amyloid [16]) within the spleen and kidney. Systemic reactive amyloidosis, particularly visualized in the spleen, kidneys, and liver, is a common sequela to sustained, chronic inflammation in other domestic and wild veterinary species [16], and should be taken into consideration as the cause in the presented case. However, because information regarding the four-eyed opossum is scarce, further structural investigations are necessary to classify the type of amyloid.

Samples of the subcutaneous mass, lung and liver were sent to the Institute of Veterinary Bacteriology, Vetsuisse Faculty, University of Bern (Switzerland) for bacteriological examination. The subcutaneous mass, lung and liver were cultured aerobically on Trypticase Soy Agar II with 5 % Sheep Blood (BD) at 37 °C in a 5 % CO_2_ atmosphere. In addition, Brolac Agar plates (ThermoFisher) were used for the subcutaneous mass and liver, whereas MacConkey Agar (ThermoFisher) was used for lung tissue at 37 °C under aerobic conditions.

After incubation of the samples from liver and mandibular mass for 48 h, more than 100 colonies of very small (approximately 1mm in diameter), convex, opaque, milky white, non-hemolytic appearance could be detected (isolate 19OD2882) in addition to a smaller quantity of β-hemolytic streptococci (*Streptococcus didelphis*) and *Pasteurella multocida*. A lower number of the same colonies was visible on sheep blood agar from the lung. No growth was visible on Brolac and MacConkey Agar.

Gram stain performed on the most abundant bacterial colonies showed Gram-positive, non-spore-forming rods, which appeared coccobacillary after culture in an aerobic atmosphere enriched with 5 % CO_2_, and slightly branched filamentous from anaerobic culture. Phenotypically, the CAMP test and catalase activity were negative. Identification of strain 19OD2882 was not possible with Maldi-Tof MS (MALDI Biotyper, Bruker using the MTB 7845 MSP Library and an in-house library) or VITEK 2 CBC card (Biomérieux).

The 16S rRNA gene was amplified using universal primers [17]. Sequence query using NCBI BLASTN, database: “16S ribosomal RNA sequences” [18] showed the highest identity with *Schaalia canis* (97.0 %). When using database “Nucleotide collection,“ 99.9 % identity was observed with accession: KF030212.1 *Actinomyces* sp. canine oral taxon 417, a thus far undescribed species, isolated from a dog in the United Kingdom.

In order to confirm the classification of this isolate as a previously undescribed species and to verify its taxonomic position, whole genome sequencing using PacBio technology was performed. gDNA was extracted following the protocol of Pitcher et al.[19] and then submitted for sequencing to the Lausanne Genomic Technologies Facility (University of Lausanne, Switzerland). Genome assembly was performed using Canu 1.9 [20] followed by polishing with arrow (SMRT Link v7.0.0, Pacific Biosciences) and circularization with Circlator 1.5.5 [21]. The genome has a size of 2.56 Mb and a GC content of 67.4 %. The genome sequence was deposited in GenBank (Accession: CP065521).

**Fig. 3:**
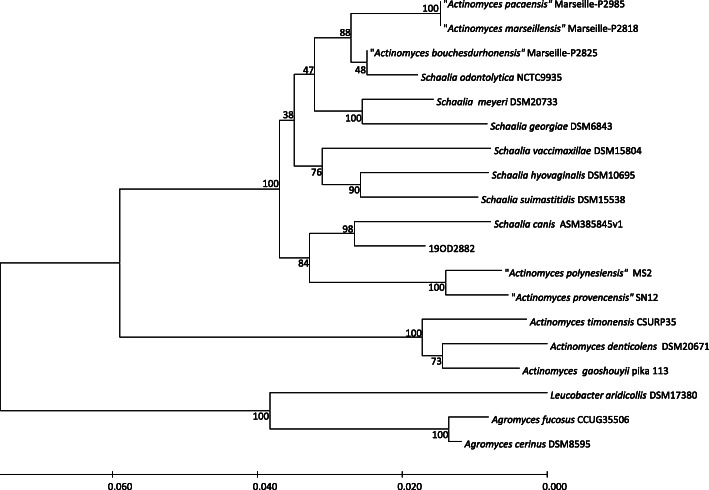
Phylogenetic tree based on 16S rRNA gene sequences. The tree was generated by the Type (Strain) Genome Server [22, 23] Tree inferred with FastME 2.1.6.1 [24] from Genome BLAST Distance Phylogeny approach (GBDP ) distances calculated from 16S rRNA gene sequences. The branch lengths are scaled in terms of GBDP distance formula *d5* [25]. The numbers above branches are GBDP pseudo-bootstrap support values. Species not validly published are indicated in quotes

**Fig. 4:**
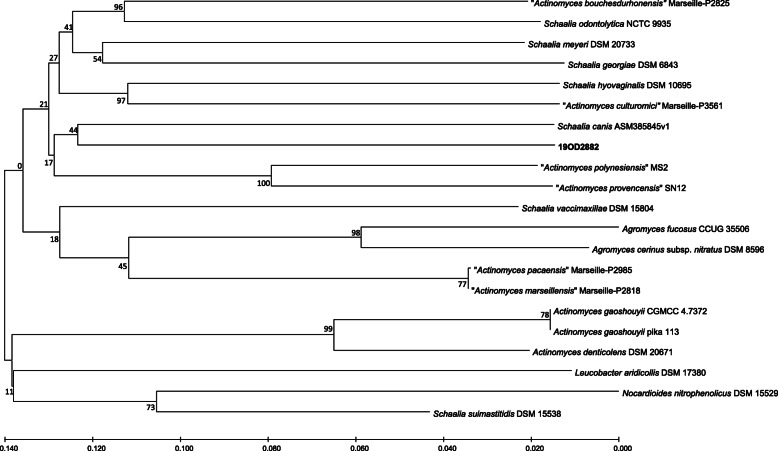
Phylogenetic tree based on the whole genome sequences. The tree was generated by the Type (Strain) Genome Server [22, 23] Tree inferred with FastME 2.1.6.1 [24] from Genome BLAST Distance Phylogeny approach (GBDP ) distances calculated from genome sequences. The branch lengths are scaled in terms of GBDP distance formula *d5* [25]. The numbers above branches are GBDP pseudo-bootstrap support values. Species not validly published are indicated in quotes

A phylogenetic tree from the 16S rRNA sequence as well as the whole genome was generated using the Type (Strain) Genome Server [22]. In both phylogenies, 19OD2882 showed the closest relationship with *Schaalia canis* (Figs. 3 and 4).

Average nucleotide identity (ANI), which is a reliable method for species differentiation, was calculated using Oat version 0.93.1 which employs the OrthoANI algorithm [26]. The cut-off for species discrimination is normally set at 95 % [27, 28]. ANI between 19OD2882 and *Schaalia canis* (NZ_RQZF01000001.1) was 71.3 % while it was 73.8 % with “*Actinomyces polynensis*” (NZ_CCXH01000001.1), both well below the cut-off. Thus, 19OD2882 clearly constitutes a previously undescribed species of *Schaalia* possibly identical to “*Actinomyces* sp. canine oral taxon 417” for which no whole genome sequence is currently reported.

The isolate was tested for antimicrobial resistance by determination of minimum inhibitory concentrations (MIC) using Sensititre plates (ANO2B, Fisher Scientific AG, Reinach, Switzerland) and following EUCAST (European Committee on Antimicrobial Susceptibility Testing) guidelines for Gram-positive anaerobes, which are recommended for all *Actinomyces* spp. [29]. Briefly, the bacteria were cultured on sheep blood agar at 37 °C under anaerobic conditions for 48 h. They were then suspended in Mueller-Hinton broth with 5 % lysed horse blood (Fisher Scientific AG, Reinach, Switzerland) at a concentration of 10^5^ CFU/ml and used for plate inoculation. The isolate was resistant to metronidazole but susceptible to all other antimicrobials tested. (Table 1).

**Table 1:** Minimal inhibitory concentrations for 19OD2882, interpretation according to EUCAST clinical breakpoints where available

Antimicrobial	MIC (mg/L)	Interpretation
Ampicillin/sulbactam (2:1 ratio)	< 0.5/0.25	S
Cefotetan	< 4	-
Imipenem	< 0.12	S
Clindamycin	< 0.25	S
Metronidazole	8	R
Ampicillin	< 0.5	S
Tetracycline	< 0.25	-
Piperacillin/ tazobactam (constant 4)	< 0.25/4	S
Penicillin	< 0.06	S
Amoxicillin/ clavulanic acid 2:1 ratio	< 0.5/0.25	S
Cefoxitin	< 1	-
Chloramphenicol	< 2	S
Piperacillin	< 4	-
Mezlocillin	< 4	-
Meropenem	< 0.5	S

## Discussion and conclusion

Lumpy jaw disease (LJD) is a well-known bacterial infection in marsupial species. The primary cause of LJD in the macropod marsupials is considered to be *Fusobacterium necorphorum*. While, *Actinomyces* spp. are capable of producing lesions in bone, their role in “jaw disease” remains undefined. However, anaerobic actinomycetes were isolated in pure culture from jaw lesions in macropods [30].

As actinomycetes are physiological inhabitants of the oral cavity and pharynx, the source of infections with these organisms tends to be endogenous, and mostly associated with trauma to the alveolar region or oral mucosa [31]. In veterinary medicine, studies mostly focus on the composition of the canine oral microbiome, in which the opportunistic pathogens *Actinomyces* and *Fusobacterium* spp. are reported to be highly abundant [32]. Oral microbiome constituents of most other veterinary species have not yet been well described. In marsupial species, *Actinomyces* spp. was recovered from 46 % of samples from the healthy, oral mucosa [30]. Given this knowledge, it can be assumed that these bacterial species also belong to the normal oral microbiome of macropods and may lead to opportunistic actinomycosis. However, according to our knowledge, little is known regarding oral flora or pathogenicity of *Actinomyces* spp. in *Philander opossum*.

*Streptococcus didelphis* was also isolated from the subcutaneous mass, liver and lung tissue of the here described gray four-eyed opossum, albeit in a lower concentration than the *Schaalia* sp.. In one report, these β-hemolytic streptococci were first found in lung, kidney, liver, spleen and skin lesions from nine opossums presenting with skin lesions, followed by sudden death [33]. Primary lesions in these animals included liver fibrosis and suppurative necrotizing dermatitis, cellulitis and myonecrosis [33]. Due to this, it cannot be entirely excluded from consideration whether *Streptococcus didelphis* may also have played a role in development of disease. However, lesions of the present case demonstrated a primarily pyogranulomatous reaction with typical Splendore-Hoeppli phenomenon associated with actinomycosis, and suggests that the novel *Schaalia* sp. was most probably the main pathogen in this case.

Furthermore, little is currently known regarding virulence traits in the genus *Actinomyces*. Whether fimbriae, peptidoglycan and biofilm production, as described in human cases, can lead to systemic involvement and death in animals, remains unclear [2].

According to 16S rRNA gene sequences, isolate 19OD2882 clearly belonged to a new species of the genus *Schaalia*, recently split from *Actinomyces* following comprehensive genetic analyses [10]. Interestingly, the 16S rRNA closely matched KF030212.1 *Actinomyces* sp. canine oral taxon 417, indicating that this bacterial species is also part of the oral microbiome of dogs.

This case presentation could be of great interest for zoo veterinarians, especially with regard to treatment options of actinomycosis in the opossum. In human medicine, actinomycosis is treated with long term antibiotics, and according to current literature, antibiotic resistance seems to not play a role. *Actinomyces* spp. do not produce beta-lactamases and often are susceptible to beta-lactams antibiotics, most notably amoxicillin and penicillin G [34]. Most strains are expected to be resistant to metronidazole, such as the presently isolated strain 19OD2882 [34, 35]. Fluoroquinolones, as applied once in this case, are not considered clinically effective against Gram-positive anaerobes including actinomycetes [29, 35].

Long-time therapy of approximately six months duration with oral clindamycin syrup (11 mg/kg twice a day) has been demonstrated to be effective in treating kangaroo lumpy jaw cases. Clindamycin capsules are also available, but have a bitter taste and are not palatable to most kangaroos [15].

This first report of a case of a *Schaalia*-infection in the gray four-eyed opossum gives an interesting insight into exotic animal bacterial diseases, and may hopefully encourage veterinarians and veterinary researchers alike to explore and expand this field further. Better knowledge about bacterial species and their pathogenicity in wildlife will be helpful to choose appropriate therapies, and to ultimately improve clinical outcomes, particularly for exotic and endangered species.

## Data Availability

The genome sequence data generated during the current study are available in the GenBank repository under Accession No. CP065521. All other data generated or analyzed during this study are included in this published article.

## Abbreviations

LJD: Lumpy Jaw Disease
ANI: Average Nucleotide Identity
EUCAST: European Committee on Antimicrobial Susceptibility Testing
MALDI-TOF-MS: Matrix-assisted laser desorption/ionization time-of-flight mass spectrometry
MIC: Minimum Inhibitory Concentration
